# Stability of single and double layer fibrillar amyloid-β oligomers

**DOI:** 10.1186/1758-2946-5-S1-P9

**Published:** 2013-03-22

**Authors:** Anna Kahler, Anselm HC Horn, Heinrich Sticht

**Affiliations:** 1 Bioinformatik, Friedrich-Alexander-Universität Erlangen-Nürnberg, Erlangen, 91054, Germany

## 

Alzheimer's disease (AD) is the most common form of dementia world-wide. The causative agent in this protein misfolding disease is the 39 to 42-residues long amyloid-β (Aβ) peptide, that aggregates into oligomers, filaments, and fibrils found in plaque deposits in the brain of AD patients [1].

Up to now a viable cure for this disease is still not available. One reason for this is Aβ's conformational flexibility and structural heterogeneity in solution paired with its aggregation tendency. This renders the determination and isolation of distinct Aβ structures experimentally challenging. Especially the soluble oligomers, that are thought to be the neurotoxic species in AD, may adopt a plethora of conformations in vivo [1].

It is known from experiment, that there exist toxic fibrillar oligomers, but the details of their topology are not yet known [2]. Thus, we used molecular dynamics simulations to investigate the structural stability of fibrillar single and ouble layer Aβ42 oligomers of different size (4-mer to 48-mer), that we constructed from the experimental structure [3] (cf Figure 1).

**Figure 1:**
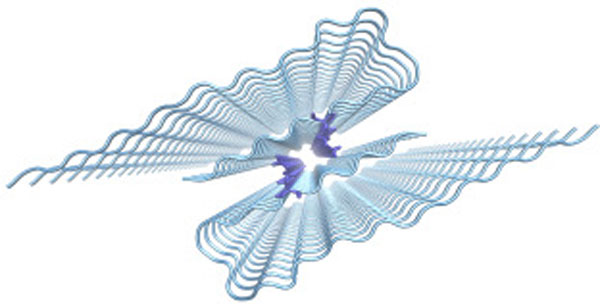
**Double layer Aβ 48-mer (Met35 in the interface in sticks representation)**.

We found that there is a clear correlation between oligomer size and preference for double layer structure: Large oligomers display an enhanced stability in double layer conformation, whereas small oligomers prefer the single layer structure. On the other hand, large single layer oligomers dissociate into smaller oligomers, while small double layer oligomers collapse or are energetically unfavorable. From our simulations we deduce that the critical number of oligomers to construct a stable Aβ double layer is in the range of 10 to 12.

In a more general picture, longitudinal growth along a single layer is limited by the increasing structural instability. Lateral growth, i.e. forming a double layer, creates stable mini-fibrils. These may act as seeds for further stable fibril growth.

## References

[B1] Haass C Selkoe DJ Soluble protein oligomers in neurodegeneration: lessons from the Alzheimer's amyloid beta-peptide Nat Rev Mol Cell Biol 2007 8 101 112 10.1038/nrm2101 17245412

[B2] Stroud JC Liu C Teng PK Eisenberg D Toxic fibrillar oligomers of amyloid-β have cross-β structure PNAS 2012 109 7717 7722 10.1073/pnas.1203193109 22547798 PMC3356606

[B3] Lührs T Ritter C Adrian M Riek-Loher D Bohrmann B Döbeli H Schubert D Riek R 3D structure of Alzheimer's amyloid-beta(1-42) fibrils PNAS 2005 102 17342 17347 10.1073/pnas.0506723102 16293696 PMC1297669

